# The impact of HMG-CoA reductase inhibitors use on the clinical outcomes in critically ill patients with COVID-19: A multicenter, cohort study

**DOI:** 10.3389/fpubh.2022.877944

**Published:** 2022-08-11

**Authors:** Khalid Al Sulaiman, Ohoud Aljuhani, Ghazwa B. Korayem, Ali F. Altebainawi, Shmeylan Al Harbi, Abdulrahman Al Shaya, Hisham A. Badreldin, Raed Kensara, Abdullah F. Alharthi, Jahad Alghamdi, Ahad Alawad, Rand Alotaibi, Abdullah Kharbosh, Hessa Al Muqati, Abdulmohsen Alhuwahmel, Mohammed Almusallam, Ghada Albarrak, Ibrahim Al Sulaihim, Bader Alanazi, Bodoor S. Al-Dosari, Ramesh Vishwakarma, Alawi S. Alsaeedi, Ghassan Al Ghamdi, Hadeel Alkofide, Hasan M. Al-Dorzi

**Affiliations:** ^1^Pharmaceutical Care Department, King Abdulaziz Medical City, Riyadh, Saudi Arabia; ^2^College of Pharmacy, King Saud bin Abdulaziz University for Health Sciences, Riyadh, Saudi Arabia; ^3^King Abdullah International Medical Research Center-King Saud Bin Abdulaziz University for Health Sciences, Ministry of National Guard—Health Affairs, Riyadh, Saudi Arabia; ^4^Saudi Critical Care Pharmacy Research (SCAPE) Platform, Riyadh, Saudi Arabia; ^5^Department of Pharmacy Practice, Faculty of Pharmacy, King Abdulaziz University, Jeddah, Saudi Arabia; ^6^Department of Pharmacy Practice, College of Pharmacy, Princess Nourah Bint Abdulrahman University, Riyadh, Saudi Arabia; ^7^Pharmaceutical Care Services, King Salman Specialist Hospital, Hail Health Cluster, Hail, Saudi Arabia; ^8^Clinical Pharmacy Department, Pharmacy College, Taif University, Taif, Saudi Arabia; ^9^Pharmaceutical Care Department, Presidency of State Security, Central Security Hospitals, Riyadh, Saudi Arabia; ^10^Pharmaceutical Care Department, King Abdulaziz University Hospital, Jeddah, Saudi Arabia; ^11^Statistics Department, European Organization for Research and Treatment of Cancer (EORTC) Headquarters, Brussels, Belgium; ^12^Intensive Care Department, King Abdulaziz Medical City, Riyadh, Saudi Arabia; ^13^College of Medicine, King Saud Bin Abdulaziz University for Health Sciences, Riyadh, Saudi Arabia; ^14^Department of Clinical Pharmacy, College of Pharmacy, King Saud University, Riyadh, Saudi Arabia

**Keywords:** COVID-19, SARS-CoV-2, statin, critically ill, intensive care units (ICUs), HMG-CoA Reductase Inhibitors, mortality, pleiotropic effect

## Abstract

**Background:**

The cardiovascular complications of Coronavirus Disease 2019 (COVID-19) may be attributed to the hyperinflammatory state leading to increased mortality in patients with COVID-19. HMG-CoA Reductase Inhibitors (statins) are known to have pleiotropic and anti-inflammatory effects and may have antiviral activity along with their cholesterol-lowering activity. Thus, statin therapy is potentially a potent adjuvant therapy in COVID-19 infection. This study investigated the impact of statin use on the clinical outcome of critically ill patients with COVID-19.

**Methods:**

A multicenter, retrospective cohort study of all adult critically ill patients with confirmed COVID-19 who were admitted to Intensive Care Units (ICUs) between March 1, 2020, and March 31, 2021. Eligible patients were classified into two groups based on the statin use during ICU stay and were matched with a propensity score based on patient's age and admission APACHE II and SOFA scores. The primary endpoint was in-hospital mortality, while 30 day mortality, ventilator-free days (VFDs) at 30 days, and ICU complications were secondary endpoints.

**Results:**

A total of 1,049 patients were eligible; 502 patients were included after propensity score matching (1:1 ratio). The in-hospital mortality [hazard ratio 0.69 (95% CI 0.54, 0.89), *P* = 0.004] and 30-day mortality [hazard ratio 0.75 (95% CI 0.58, 0.98), *P* = 0.03] were significantly lower in patients who received statin therapy on multivariable cox proportional hazards regression analysis. Moreover, patients who received statin therapy had lower odds of hospital-acquired pneumonia [OR 0.48 (95% CI 0.32, 0.69), *P* < 0.001], lower levels of inflammatory markers on follow-up, and no increased risk of liver injury.

**Conclusion:**

The use of statin therapy during ICU stay in critically ill patients with COVID-19 may have a beneficial role and survival benefit with a good safety profile.

## Introduction

Since the spread of the Severe Acute Respiratory Syndrome Corona Virus 2 (SARS-CoV-2), leading to Coronavirus Infectious Disease 2019 (COVID-19) in 2019, over six million people have died worldwide (1). Although the mortality of patients with COVID-19 is mainly due to respiratory-related complications, growing evidence shows increased morbidity and mortality related to multiorgan failure, including heart and kidney failure (2–5). Evidence suggests that critically ill patients with COVID1-9 may have an overproduction of early response pro-inflammatory cytokines, which results in a systemic hyperinflammatory state that may contribute to developing acute respiratory distress syndrome (ARDS) (6, 7). Hypoxia plays a major role in COVID-19 mortality (8, 9), and can trigger cardiorespiratory compensation which may fail causing lactic acid elevation, cardiovascular failure and death (10, 11). Moreover, pre-existing cardiovascular diseases (CVD) or cardiovascular risk factors in subjects with COVID-19 can confer a higher risk of poor prognosis and increased mortality (12–14).

In non-COVID-19 patients, statin therapy has been associated with lower Cardiovascular risk and mortality (15). Statins have pleiotropic anti-inflammatory, antithrombotic and immunomodulatory effects which may decrease the endothelial dysfunction and inflammatory dysregulation in patients with COVID-19 (16–18). Part of the anti-inflammatory effects of statins is ultimately to reduce inflammatory markers such as C-reactive proteins (CRP) (19). Moreover, statin therapy has been reported to have antiviral activity through immunomodulation and viral replication suppression (20). These effects of statins suggest that they might have a promising role in indirectly improving the clinical outcomes in patients with COVID-19 (21). This may be attributed to the improvement in endothelial dysfunction suppressing the damage that may be caused by microvascular, and macrovascular thrombosis and cytokine storm (22, 23); thus, reducing cardiovascular complications in COVID-19 patients.

The clinical evidence about the benefit of statins is inconsistent (24, 25). Multiple studies reported a decrease in mortality and inflammatory response in patients with COVID-19 using statin therapy (24, 25). In contrast, a systematic review and national observational study respectively showed no improvement in hospital outcomes and even an increase in mortality (26, 27). Nonetheless, most previous studies were performed in hospitalized patients with mild to severe COVID-19, while only few studies investigated statins in critically ill patients with COVID-19 (21). Thus, this study aimed to investigate the impact of statins use on the clinical outcomes in critically ill patients with COVID-19.

## Methods

### Study design

This was a multicenter, retrospective cohort study including adult critically ill patients with confirmed COVID-19 who were admitted to the intensive care units (ICUs) from March 01, 2020, until March 31, 2021. Eligible patients were then classified into two groups based on statin therapy use during ICU stay (non-statin vs. statin users). Statins were prescribed in the ICU as part of the medication reconciliation process if they had been prescribed in the pre-ICU period or were initiated in the ICU for various indications at the discretion of the ICU treating team and stopped based on physicians' clinical judgment. The King Abdullah International Medical Research Center (KAIMRC) authorized the study in January 2021 (Reference number: NRC21R/015/R). Due to the study's retrospective observational nature, informed consent from study participants was waived.

### Study participants

**Figure 1:**
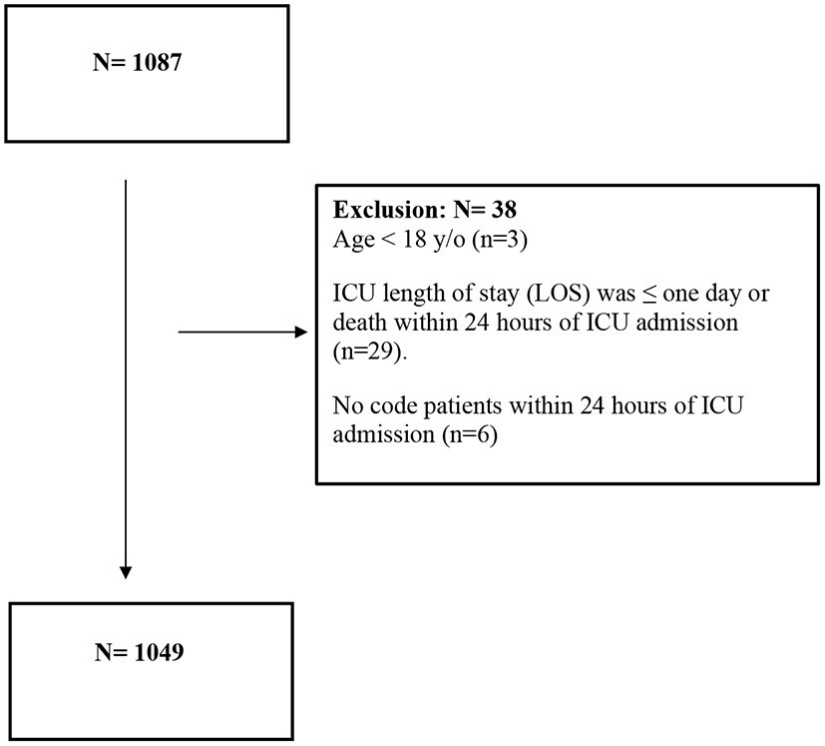
Critically ill patients with COVID-19 flowchart.

Adult (age 18 years and older) critically ill patients who were admitted to ICUs at four centers with confirmed COVID-19, regardless of their comorbid conditions, were included in the study. Reverse Transcriptase-Polymerase Chain Reaction (RT-PCR) nasopharyngeal and/or throat swabs were used to diagnose COVID-19. Patients were excluded if they had an ICU length of stay (LOS) of 1 day or less, died within 24 h of admission, or were designated a “Do-Not-Resuscitate” status within 24 h of their ICU admission (Figure 1). All patients were followed until they were discharged from the hospital or died during the hospital stay, whichever occurred first.

### Study setting

This study was conducted at four centers in Saudi Arabia: King Abdulaziz Medical City (Riyadh), King Abdullah bin Abdulaziz University Hospital (KAAUH) (Riyadh), King Abdulaziz University Hospital (Jeddah), and King Salman Specialist Hospital (Hail). We included both tertiary and secondary centers. The selection of these centers was based on the geographic distribution, availability of electronic records and the center's willingness to participate in this national project. King Abdulaziz Medical City (Riyadh), a tertiary care center that includes more than 1500 beds and more than nine ICU units, was the primary site for this multicenter retrospective study.

### Data collection

Each patient's data were collected and handled using RREDCap^®^ software hosted by the King Abdullah International Medical Research Center (KAIMRC). We collected demographic data, comorbidities, vital signs, laboratory tests, severity scores (i.e., Sequential Organ Failure Assessment (SOFA), Nutrition Risk in Critically ill (*NUTRIC*), and Acute Physiology and Chronic Health Evaluation II (APACHE II) scores), Glasgow Coma Score (GCS), receipt of mechanical ventilation (MV) and MV parameters (e.g., highest FiO_2_ requirement, lowest PaO_2_/FiO_2_ ratio), use of prone positioning, and acute kidney injury within 24 h of ICU admission. Moreover, liver function tests (LFTs), coagulation profile (i.e., fibrinogen, D-dimer, INR, aPTT), renal profile, and inflammatory markers (e.g., ferritin, and procalcitonin) within 24 h of ICU admission were collected. For the eligible patients, HMG-CoA Reductase Inhibitors (statin therapy) (type and dose), tocilizumab and corticosteroids use were recorded. We also included information on the use of inotropes and vasopressors during the first 24 h of ICU admission, as well as information on the use of nephrotoxic drugs and DVT prophylaxis during ICU admission. If administering oral tablet was not feasible, then statin tablet was crushed and given to the patient through the feeding tube, according to hospital policy.

### Outcomes

In-hospital mortality was the primary endpoint. Other outcomes such as 30-day mortality, hospital LOS, ICU LOS, receipt of MV, ventilator-free days (VFDs) at 30 days, and ICU-acquired complication**s** (new-onset atrial fibrillation, respiratory failure, hospital-acquired pneumonia [(bacterial or fungal), secondary fungal infection, acute kidney injury, and liver injury] were considered secondary endpoints (Supplementary material 1).

### Statistical analysis

We reported continuous data as mean and standard deviation (SD), or median with the lower quartile (Q1) and upper quartile (Q3), as appropriate, and categorical variables as number and percentage. The baseline variables of the two study groups were compared (statin and non-statin users). We employed the Chi-square or Fisher's exact test categorical data. On the other hand, we used the student *t*-test for normally distributed continuous data and the Mann-Whitney *U*-test for non-normally distributed continuous variables. The normality assumptions were assessed for all numerical variables using graphical representation (i.e., histograms and Q-Q plots) and statistical tests (i.e., Shapiro–Wilk test). Hosmer-Lemeshow goodness-of-fit test was used for model fit assessment.

Propensity score matching (Proc PS match; SAS, Cary, NC) was used to match patients who received statin medication (active group) to patients who did not (control group) based on patient's age and baseline illness severity scores (APACHE II and SOFA scores) within 24 h of ICU admission. A greedy nearest neighbor matching approach was utilized, such that one patient who received statin medication (active) was paired with one patient who did not (control), resulting in the least within-pair difference among all available pairs of treated patients. The difference in the logits of the propensity scores for pairs of patients from the two groups was matched only if it was ≤0.5 times the pooled estimate of the standard deviation. To validate the propensity score model, baseline characteristics for the matched cohort were compared between the two groups.

By considering the PS score as one of the covariates in the model, regression analysis was done based on the patient's age and baseline severity scores (APACHE II and SOFA) within 24 h of ICU admission. For the 30-day and in-hospital mortality, multivariable cox proportional hazards regression analyses were performed, and the proportionality assumption was assessed before fitting the cox model. Visual assessment was performed to assess the assumption by plotting log(-log) plot and by testing the correlation of scaled Schoenfeld residuals with rank-ordered time. Multivariable and negative binomial regression analysis were used for all other outcomes considered in this study. The hazard ratio (HR), odds ratios (OR), or estimates with the 95% confidence intervals (CI) were reported as appropriate. Since the patients in our study were not selected randomly, no imputation was performed for missing data. All statistical analyses were performed using SAS version 9.4 and a *P*-value of < 0.05 was considered statistically significant.

## Results

A total of 1,049 patients met our inclusion criteria. Of these patients, 279 (26.6%) received statins during their ICU stay. After propensity score matching, 502 patients were included (1:1 ratio) according to the selected criteria. Most of the patients (86.4%) received statins as a continuation due to underlying coexisting illness [e.g., dyslipidemia (DLP), ischemic heart disease (IHD)]. Only 20 patients (8.6%) required statin discontinuation during ICU stay. The most common cause for statin discontinuation was rhabdomyolysis (nine patients), followed by liver damage (eight patients). Atorvastatin (81.3%) with a median daily dose of 20 mg was the most often used statin, followed by rosuvastatin (14.1%) with a median daily dose of 20 mg.

### Demographic and clinical characteristics

Most of the included patients in both arms were males (68.9%) with a mean age of 61.6 years (SD ± 14.8). Diabetes mellitus (59.0%) was the predominant underlying comorbidity, followed by hypertension (55.2%) and dyslipidemia (19.3%). There were some notable differences between the two groups before propensity score matching. Following the propensity score (PS) matching, most of the baseline and demographic characteristics were similar between the two groups. Summary of the patients' baseline characteristics is available in Table 1.

**Table 1:** Summary of demography and baseline characteristics.

	**Before propensity score (PS) adjustment**				**After propensity score (PS) adjustment**			
	**Overall (*N* = 1,049)**	**Control (*N* = 770)**	**Statin (*N* = 279)**	***P*-value**	**Overall (502)**	**Control (*N* = 251)**	**Statin (*N* = 251)**	***P*-value**
Age (years), mean (SD)	61.6 (14.79)	60.2 (15.07)	65.5 (13.25)	<0.0001^*^	65.2 (13.86)	64.9 (14.31)	65.4 (13.41)	0.7258^*^
Gender—Male, *n* (%)	698 (68.9)	518 (69.8)	180 (66.4)	0.3021^∧∧^	327 (65.8)	163 (64.9)	164 (66.7)	0.6850^∧∧^
Weight (kg), mean (SD)	81.2 (18.90)	80.8 (18.38)	82.4 (20.22)	0.6326^∧^	59.6 (51.50, 66.01)	59.7 (52.41, 66.01)	58.7 (50.54, 65.10)	0.0991^∧^
APACHE II score, median (Q1,Q3)	14.0 (9.00, 23.00)	15.0 (9.00, 25.00)	13.0 (10.00, 20.00)	0.0846^∧^	12.5 (9.00, 19.00)	12.0 (8.00, 17.00)	13.0 (10.00, 20.00)	0.2449^∧^
SOFA score, median (Q1,Q3)	5.0 (3.00, 8.00)	5.0 (3.00, 8.00)	5.0 (3.00, 8.00)	0.7479^∧^	5.0 (3.00, 7.00)	5.0 (3.00, 7.00)	5.0 (3.00, 8.00)	0.3806^∧^
Systemic corticosteroids use during ICU, *n* (%)	155 (15.2)	96 (12.8)	59 (21.6)	0.0005^∧^	411 (82.5)	204 (81.3)	207 (83.8	0.4571^∧∧^
Tocilizumab use, *n* (%)	367 (35.9)	266 (35.6)	101 (37.0)	0.6724^∧∧^	183 (36.7)	88 (35.1)	95 (38.5)	0.4311^∧∧^
Proning, *n* (%)	302 (30.5)	240 (33.1)	62 (23.5)	0.0038^∧∧^	140 (28.1)	80 (31.9)	60 (24.3)	0.06^∧∧^
Estimated Glomerular Filtration Rate (eGFR) baseline (mL/min/1.73m2), median (Q1, Q3)	76.0 (45.00, 97.00)	76.5 (47.00, 98.00)	73.0 (41.00, 97.00)	0.2037^∧^	75.0 (47.00, 97.00)	76.0 (54.00, 99.00)	73.0 (40.00, 96.00)	0.0801^∧^
Acute Kidney Injury (AKI) Within 24 h of ICU admission, *n* (%)	251 (25.3)	186 (25.6)	65 (24.5)	0.7349^∧∧^	115 (23.4)	56 (22.7)	59 (24.1)	0.7118^∧∧^
Mechanical ventilation within 24 h of ICU admission, *n* (%)	719 (70.5)	511 (68.3)	208 (76.5)	0.0116^∧∧^	356 (71.5)	166 (66.1)	190 (76.9)	0.0077^∧∧^
Inotropes/vasopressors use within 24 h of admission)	222 (22.2)	153 (20.9)	69 (25.7)	0.1003^∧∧^	110 (22.2)	46 (18.5)	64 (25.9)	0.0462^∧∧^
Lactic acid baseline (mmol/L), median (Q1,Q3)	1.7 (1.26, 2.32)	1.7 (1.22, 2.40)	1.7 (1.30, 2.20)	0.9041^∧^	1.6 (1.28, 2.34)	1.6 (1.23, 2.45)	1.6 (1.30, 2.23)	0.8520^∧^
Platelets count baseline (10^9^/L), median (Q1,Q3)	241.0 (183.00, 311.00)	239.5 (182.00, 309.00)	243.0 (186.00, 316.00)	0.6098^∧^	247.0 (186.00, 313.00)	254.0 (186.50, 313.00)	243.0 (186.00, 313.00)	0.9822^∧^
Total WBC baseline (10^9^/L), median (Q1,Q3)	16.1 (11.05, 23.00)	16.5 (11.07, 24.24)	15.1 (11.00, 20.25)	0.0142^∧^	15.8 (11.00, 22.20)	16.5 (11.00, 23.70)	15.2 (11.00, 20.60)	0.0983^∧^
International normalized ratio (INR), median (Q1,Q3)	1.1 (1.03, 1.21)	1.1 (1.04, 1.23)	1.1 (1.00, 1.15)	0.8492^*^	1.1 (1.02, 1.18)	1.1 (1.03, 1.19)	1.1 (1.01, 1.15)	0.0088^∧^
activated partial thromboplastin time (aPTT) baseline (sec), median (Q1,Q3)	30.3 (27.00, 33.90)	30.9 (27.00, 34.20)	29.3 (26.30, 33.05)	0.0020^∧^	29.7 (26.70, 33.40)	30.1 (26.75, 34.00)	29.3 (26.40, 33.00)	0.1387^∧^
Total bilirubin (μmol/L), median (Q1,Q3)	9.8 (6.80, 14.00)	10.0 (6.85, 14.00)	9.2 (6.30, 13.60)	0.2141^∧^	9.1 (6.60, 13.20)	9.0 (6.60, 13.00)	9.3 (6.40, 13.45)	0.8668^∧^
Albumin baseline (gm/L), median (Q1,Q3)	32.0 (28.00, 36.00)	32.0 (28.00, 37.00)	32.4 (29.00, 35.00)	0.8750^∧^	32.0 (29.00, 35.00)	32.0 (27.00, 36.00)	32.5 (29.00, 35.00)	0.1863^∧^
Creatine phosphokinase (CPK) baseline (U/l), median (Q1,Q3)	173.5 (75.00, 426.00)	183.0 (78.00, 472.00)	153.0 (63.00, 333.00)	0.0069^∧^	153.0 (69.00, 347.00)	146.5 (70.50, 352.50)	160.0 (69.00, 344.00)	0.9775^∧^
C-reactive protein (CRP) baseline (mg/l), median (Q1,Q3)	130.0 (65.22, 197.50)	126.8 (53.52, 194.95)	135.0 (82.00, 206.00)	0.0245^∧^	124.0 (67.00, 196.20)	117.0 (30.00, 191.00)	136.0 (85.00, 203.00)	0.0013^∧^
Fibrinogen level baseline (gm/l), median (Q1,Q3)	5.4 (3.77, 7.15)	5.4 (3.56, 7.18)	5.6 (4.21, 7.05)	0.3941^∧^	5.3 (3.88, 7.04)	5.2 (3.43, 6.92)	5.5 (4.20, 7.36)	0.2236^∧^
D-dimer level baseline (mg/l), median (Q1,Q3)	1.3 (0.71, 3.34)	1.4 (0.72, 3.58)	1.2 (0.63, 2.51)	0.0443 [∧]	1.2 (0.66, 2.72)	1.2 (0.70, 2.89)	1.2 (0.62, 2.53)	0.3530^∧^
Ferritin level baseline (ug/l), median (Q1,Q3)	782.5 (383.60, 1,647.00)	867.9 (436.50, 1,650.00)	570.5 (286.70, 1,137.50)	<0.0001^∧^	888.2 (384.30, 1,650.00)	936.9 (466.60, 1,650.00)	732.3 (332.00, 2,118.00)	0.1611^∧^
PaO2/FiO2 ratio within 24 h of admission, median (Q1,Q3)	83.1 (60.28, 136.10)	85.0 (60.00, 142.40)	80.5 (63.58, 124.30)	0.5852^∧^	79.5 (60.00, 125.30)	78.1 (58.00, 125.00)	81.5 (61.80, 125.60)	0.3576^∧^
Pharmacological DVT prophylaxis use during ICU stay, *n* (%)	219 (24.6)	146 (23.0)	73 (28.9)	0.0656^∧∧^	468 (94.5)	232 (93.5)	236 (95.5)	0.3277^∧∧^
Patient received nephrotoxic drugs/material during ICU stay	823 (81.6)	576 (78.0)	247 (91.1)	<0.0001^∧∧^	424 (85.8)	202 (81.1)	222 (90.6)	0.0025^∧∧^
Comorbidity, *n* (%)								
Atrial fibrillation (A Fib)	28 (2.7)	15 (2.0)	13 (4.8)	0.0170^∧∧^	16 (3.2)	3 (1.2)	13 (5.3)	0.0101^∧∧^
Heart failure	85 (8.3)	55 (7.4)	30 (11.0)	0.0627^∧∧^	44 (8.8)	16 (6.4)	28 (11.3)	0.0511^∧∧^
Hypertension (HTN)	564 (55.2)	354 (47.3)	210 (76.9)	<0.0001^∧∧^	311 (62.4)	116 (46.2)	195 (78.9)	<0.0001^∧∧^
Diabetes mellitus (DM)	602 (59.0)	406 (54.3)	196 (71.8)	<0.0001^∧∧^	318 (63.9)	138 (55.0)	180 (72.9)	<0.0001^∧∧^
Dyslipidemia (DLP)	197 (19.3)	70 (9.4)	127 (46.5)	<0.0001^∧∧^	139 (27.9)	27 (10.8)	112 (45.3)	<0.0001^∧∧^
Ischemic heart disease (IHD)	84 (8.2)	54 (7.2)	30 (11.0)	0.0524^∧∧^	44 (8.8)	20 (8.0)	24 (9.7)	0.4918^∧∧^
Chronic kidney disease (CKD)	113 (11.1)	66 (8.8)	47 (17.2)	0.0002^∧∧^	63 (12.7)	17 (6.8)	46 (18.6)	<0.0001^∧∧^
Cancer	25 (2.4)	19 (2.5)	6 (2.2)	0.7541^∧∧^	14 (2.8)	8 (3.2)	6 (2.4)	0.6088^∧∧^
Deep Vein Thrombosis (DVT)	7 (0.7)	6 (0.8)	1 (0.4)	0.4551^**^	1 (0.2)	0 (0.0)	1 (0.4)	0.3129^**^
Pulmonary embolism	7 (0.7)	5 (0.7)	2 (0.7)	0.9125^**^	3 (0.6)	1 (0.4)	2 (0.8)	0.5531^**^
Liver disease (any type)	3 (0.3)	3 (0.4)	0 (0.0)	0.2947^**^	12 (2.4)	5 (2.0)	7 (2.8)	0.5401^∧∧^
Stroke	56 (5.5)	28 (3.7)	28 (10.3)	<0.0001^∧∧^	31 (6.2)	8 (3.2)	23 (9.3)	0.0047^∧∧^

^*^T-test/^∧^Wilcoxon rank sum test is used to calculate the P-value.

PS, Propensity score.

^∧∧^Chi square/^**^Fisher's Exact teat is used to calculate P-value.

### 30-day and in-hospital mortality

**Figure 2:**
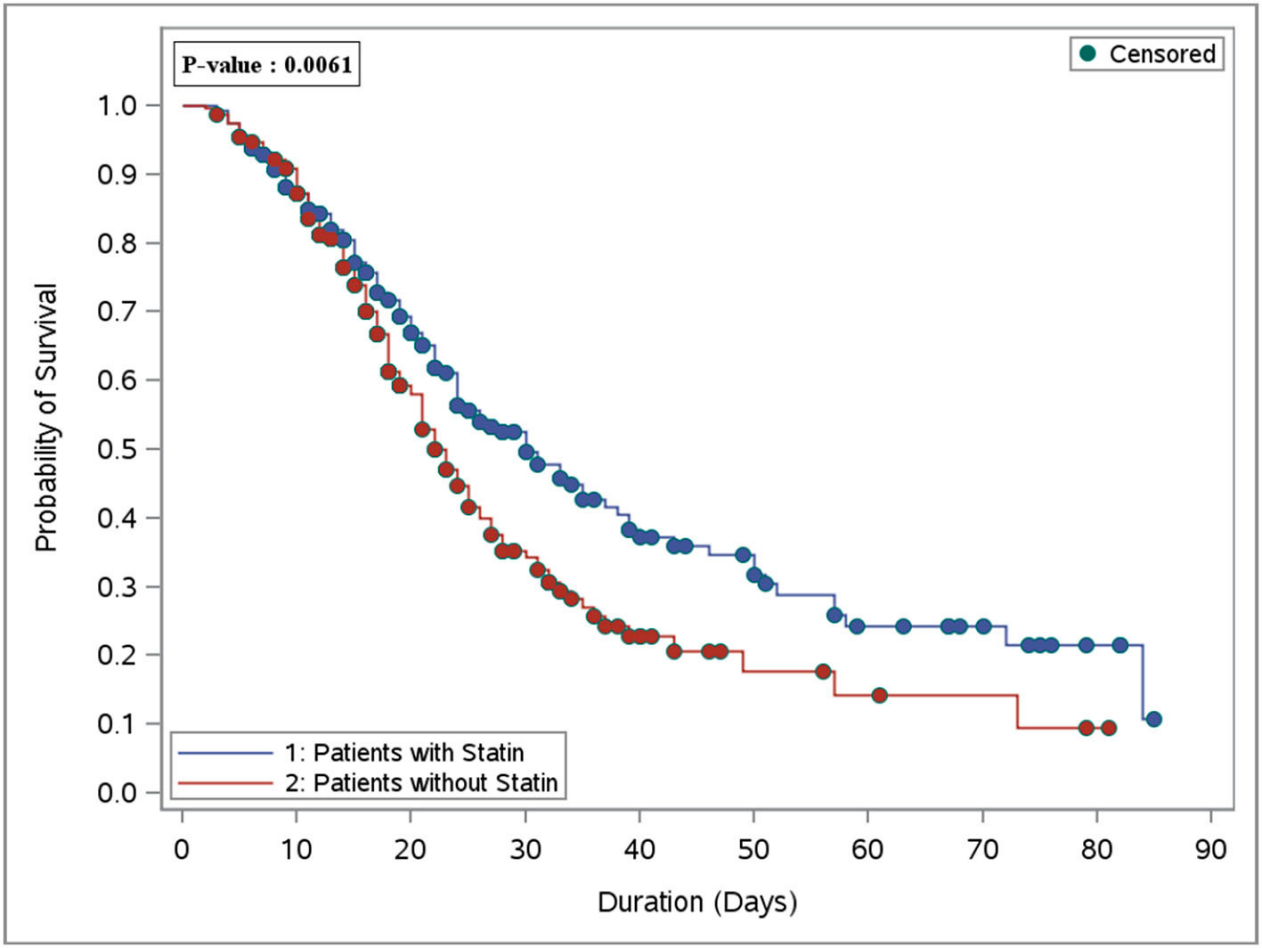
Overall survival plot during the hospital stay after PS matching comparing patients who received statin therapy (251 patients) vs. the control group (non-statin; 251 patients).

In the crude analysis, there was no significant difference in the in-hospital mortality (46.8 vs. 53.0%, *P* = 0.17) or 30-day mortality (44.2 vs. 49.2%, *P* = 0.27) between the statin and the control group, respectively (Table 1). However, using the cox proportional hazards regression analysis, patients who received statins had a lower in-hospital mortality [HR 0.69 (95% CI 0.54, 0.89), *P* = 0.004] as well as 30-day mortality [HR 0.75 (95% CI 0.58, 0.98), *P* = 0.03] compared with the control group (Table 2). In the prespecified subgroup analysis (Tables 3, 4), in patients who were on statin pre-ICU admission (Chronic use), the in-hospital mortality was lower in the statin group (HR 0.79; 95% CI 0.59, 1.04; *P* = 0.09); however, it did not reach to a statistical significant difference. The overall survival probabilities were higher during hospital stay among patients who received statin therapy before and after propensity score-matching (Figure 2).

**Table 2:** The outcomes of critically ill patients with COVID-19 after propensity score matching.

**Outcomes**	**Number of outcomes/** **total number of patients**			**Hazard ratio (HR) (95%CI)**	***P*-value^$^**
	**Control**	**Statin**	***P*-value**		
30-day mortality, *n* (%)^Δ^	122/248 (49.2)	111/251 (44.2)	0.27^∧∧^	0.75 (0.58, 0.98)	0.03
In-hospital mortality, *n* (%)^Δ^	132/249 (53.0)	117/250 (46.8)	0.17^∧∧^	0.69 (0.54, 0.89)	0.004
				**Beta coefficient (estimates)** **(95%CI)**	***P*** **-value** ^$*^
Ventilator free days, mean (SD)	10.2 (±12.3)	10.6 (±12.2)	0.61^∧^	0.03 (-0.38, 0.45)	0.87
ICU Length of Stay (LOS) (days), median (Q1, Q3)^&^	9.0 (5.0, 14.0)	11.0 (6.0, 19.0)	0.03^∧^	0.24 (0.07, 0.42)	0.007
Hospital Length of Stay (days), median (Q1, Q3)^&^	17.0 (12.0, 28.0)	20.0 (13.0, 33.0)	0.03^∧^	0.39 (0.21, 0.58)	<0.0001

Δ The denominator of the percentage is the total number of patients.

& Denominator is patients who survived.

∧ Wilcoxon rank-sum test is used to calculate the P-value.

∧∧ Chi-square test is used to calculate the P-value.

$ Cox proportional hazards regression analysis used to calculate HR and p-value.

$* Generalized linear model is used to calculate estimates and p-value.

**Table 3:** Subgroup analysis—regression analysis for the outcomes after PS adjustment (new initiation of statin).

**Outcomes**	**Number of outcomes/** **total number of patients**			**Hazard ratio (HR) (95%CI)**	***P*-value^$^**
	**Control**	**Statin**	***P*-value**		
30-day mortality, *n* (%)^Δ^	19 (51.4)	7 (20.6)	0.007	0.58 (0.23, 1.44)	0.24
In-hospital mortality, *n* (%)^Δ^	19 (54.3)	10 (29.4)	0.04^∧∧^	0.69 (0.29, 1.62)	0.40
			***P*** **-value** ^∧^	**Beta coefficient (estimates) (95%CI)**	***P*** **-value** ^$*^
Ventilator free days, mean (SD)	7.1 (10.71)	14.0 (11.54)	0.01	0.25 (−0.71, 1.21)	0.60
ICU Length of Stay (days), median (Q1, Q3)^&^	18.5 (9.0, 27.5)	11.0 (6.0, 17.0)	0.25	−0.15 (−0.56, 0.27)	0.48
Hospital Length of Stay (days), median (Q1, Q3)^&^	32.5 (13.0, 57.5)	16.5 (11.0,28)	0.15	−0.30 (−0.78, 0.18)	0.23
**Complication (s) during ICU stay**			***P*** **-value**	**Odds ratio (OR) (95%CI)**	***P*** **-value** ^$**^
Respiratory failure required MV, *n* (%)^$$^	3/5 (60.0)	6/14 (42.8)	0.63^**^	0.73 (0.06, 8.21)	0.80
New onset Afib., *n* (%)^Δ^	7 (18.9)	3 (8.8)	0.22^**^	0.64 (0.14, 2.96)	0.56
Acute kidney injury, *n* (%)^Δ^	15 (40.5)	8 (23.5)	0.13^∧∧^	0.79 (0.25, 2.53)	0.69
Liver injury, *n* (%)^Δ^	3 (8.1)	0 (0.0)	0.08^**^	NC	NC
Hospital acquired pneumonia, *n* (%)^Δ^	17 (45.9)	9 (26.5)	0.08^∧∧^	0.41 (0.14, 1.19)	0.10
Secondary fungal infection, *n* (%)^Δ^	5 (20.0)	5 (27.8)	0.55^**^	2.02 (0.44, 9.16)	0.35

Δ Denominator of the percentage is the total number of patients.

& Denominator is patients who survived.

∧ Wilcoxon rank sum test is used to calculate the P-value.

∧∧ Chi-square test is used to calculate the P-value/^**^Fisher Exact test is used to calculate the P-value.

$ Cox proportional hazards regression analysis used to calculate HR and p-value.

$* Negative binomial regression is used to calculate estimates and p-value.

$** Logistic regression is used to calculate the OR and p-value.

$$ Denominator of the percentage is non-mechanically ventilated patients with 24 h of ICU admission.

**Table 4:** Subgroup analysis—regression analysis for the outcomes after PS adjustment (chronic use of statin only).

**Outcomes**	**Number of outcomes/** **total number of patients**			**Hazard ratio (HR) (95%CI)**	***P*-value^$^**
	**Control**	**Statin**	***P*-value**		
30-day mortality, *n* (%)^Δ^	97 (46.0)	99 (48.3)	0.63	0.93 (0.69, 1.23)	0.59
In-hospital mortality, *n* (%)^Δ^	107 (51.2)	99 (48.5)	0.59	0.79 (0.59, 1.04)	0.09
			***P*** **-value** ^∧^	**Beta coefficient (estimates) (95%CI)**	***P*** **-value** ^$*^
Ventilator free days, mean (SD)	10.9 (12.41)	10.4 (12.39)	0.69	−0.06 (−0.52, 0.39)	0.78
ICU Length of Stay (days), median (Q1,Q3)^&^	9.5 (5.0, 15.5)	11.0 (6.0, 20.0)	0.23	0.05 (−0.17, 0.25)	0.68
Hospital Length of Stay (days), median (Q1,Q3)^&^	17.5 (11.0, 28.0)	21.0 (12.0, 34.0)	0.10	0.26 (0.03, 0.48)	0.02
**Complication (s) during ICU stay**			***P*** **-value** ^∧∧^	**Odds ratio (OR) (95%CI)**	***P*** **-value** ^$**^
Respiratory Failure Required MV, *n* (%)^$$^	25/58 (43.1)	23/46 (50.0)	0.48	1.09 (0.48, 2.47)	0.83
New onset Afib., *n* (%)^Δ^	23/215 (10.7)	32/215 (14.8)	0.19	1.35 (0.76, 2.41)	0.31
Acute kidney injury, *n* (%)^Δ^	78 (36.6)	85 (41.3)	0.33	1.21 (0.81, 1.79)	0.35
Liver injury, *n* (%)^Δ^	20 (9.4)	18 (8.7)	0.82	0.89 (0.46, 1.76)	0.75
Hospital acquired pneumonia, *n* (%)^Δ^	100 (46.9)	50 (24.3)	<0.0001	0.37 (0.24, 0.56)	<0.0001
Secondary fungal infection, *n* (%)^Δ^	35 (21.0)	37 (32.7)	0.03	1.93 (1.12, 3.33)	0.02

Δ Denominator of the percentage is the total number of patients.

& Denominator is patients who survived.

∧ Wilcoxon rank sum test is used to calculate the P-value.

∧∧ Chi-square test is used to calculate the P-value/^**^Fisher Exact test is used to calculate the P-value.

$ Cox proportional hazards regression analysis used to calculate HR and p-value.

$* Negative binomial regression is used to calculate estimates and p-value.

$** Logistic regression is used to calculate the OR and p-value.

$$ Denominator of the percentage is non-mechanically ventilated patients with 24 h of ICU admission.

### Ventilator free days and length of stay

During the ICU stay, the mean VFD was 10.6 days (±12.2) in patients who received statin therapy, and 10.2 days (±12.3) in the control group; however, it was not significant between the groups [beta coefficient: 0.03 (95% CI−0.38, 0.45), *P* = 0.87]. On the other hand, among those who survived during ICU stay, we observed that critically ill patients who received statin therapy had a longer ICU LOS and hospital LOS [beta coefficient of 0.24 (95% CI 0.07, 0.42), *P* = 0.007], and [beta coefficient 0.39 (95% CI 0.21, 0.58), *P* < 0.0001, respectively; Table 2].

### Complications during ICU stay

Patients who received statins had statistically significantly lower odds of hospital-acquired pneumonia (bacterial or fungal) [OR 0.48 (95% CI 0.32, 0.69), *P* = 0.001]. On the other hand, statin users had higher odds for secondary fungal infection [OR 2.48 (95% CI 1.44, 4.24), *P* = 0.001] compared with the control group. Other complications during ICU stay were reported in Table 5. Subgroup analysis for those who were on chronic statins showed the same findings in term of lower odds of hospital-acquired pneumonia [OR 0.37 (95% CI 0.24, 0.56), *P* = 0.001] and higher odds for secondary fungal infection [OR 1.93 (95% CI 1.12, 3.33), *P* = 0.02; Table 4].

**Table 5:** The ICU complications of critically ill patients with COVID-19 during stay.

**Outcomes**	**Number of outcomes/** **total number of patients**			**Hazard ratio (HR) (95%CI)**	***P*-value^$^**
	**Control**	**Statin**	***P*-value**		
Respiratory failure requiring MV, *n* (%)^$*^	47/85 (55.3)	28/58 (48.3)	0.41^∧∧^	0.80 (0.40, 1.59)	0.53
New onset atrial fibrillation, *n* (%)Δ^∧^	25/248 (10.1)	33/238 (13.9)	0.19^∧∧^	1.44 (0.83, 2.50)	0.21
Acute kidney injury, n (%)Δ	98/251 (39.0)	100/251 (39.8)	0.85^∧∧^	1.03 (0.72, 1.48)	0.87
Liver injury, n (%)Δ	32/251 (12.8)	19/251 (7.6)	0.05^∧∧^	0.55 (0.30, 1.01)	0.05
Hospital acquired pneumonia, n (%)Δ	101/251 (40.2)	61/251 (24.3)	<0.001^∧∧^	0.48 (0.32, 0.69)	<0.001
Secondary fungal infection, n (%)Δ	29/180 (16.1)	44/137 (32.1)	0.001**	2.48 (1.44, 4.24)	0.001
**Follow-up markers (highest during ICU stay)**			***P*** **-value** ^∧^	**Beta coefficient (estimates) (95%CI)**	***P*** **-value** ^$*^
Ferritin level (ug/l), Median (Q1, Q3) Δ	936.9 (466.6, 1,650.0)	732.3 (332.0, 2,132.1)	0.18	0.18 (−0.06, 0.42)	0.14
D-dimer level (mg/l), Median (Q1, Q3) Δ	4.4 (1.98, 19.6)	3.0 (1.36, 8.15)	<0.001	−3.00 (−3.40, −2.60)	<0.001
Procalcitonin level (ng/ml), Median (Q1, Q3) Δ	0.45 (0.13, 1.76)	0.49 (0.13, 1.58)	0.77	−1.46 (−2.01, −0.91)	<0.001
Creatine phosphokinase (CPK) level (U/l), Median (Q1, Q3) Δ	180.0 (71.0, 544.0)	180.0 (84.0, 540.0)	0.81	−0.07 (−0.37, 0.23)	0.65
Total WBC count (109/L), Median (Q1, Q3) Δ	16.5 (11.0, 23.7)	15.3 (11.0, 20.6)	0.13	−0.09 (−0.19, −0.005)	0.04

$* Denominator of the percentage is non-mechanically ventilated patients with 24 h of ICU admission.

^Δ∧^Denominator of the percentage is non-atrial fibrillation as comorbidity.

^Δ^The denominator of the percentage is the total number of patients.

∧∧ Chi-square test is used to calculate the P-value/^**^Fisher's Exact teat is used to calculate P-value.

>∧ Wilcoxon rank-sum test is used to calculate the P-value.

$ Logistic regression is used to calculate the OR and p-value.

$* Generalized linear model is used to calculate estimates and p-value.

### Follow-up biomarkers during ICU stay

The follow-up biomarkers such as D-dimer (*p*-value < 0.001), procalcitonin (*p*-value < 0.001), and total WBC count (*p*-value 0.04) were significantly lower during ICU stay in patients who received statins compared with the control group. In contrast, CPK and ferritin levels were similar among the groups, as described in Table 5.

## Discussion

To the best of our knowledge, this multicenter cohort study is one of few studies that evaluated statins' clinical and safety outcomes in critically ill patients with COVID-19 using propensity score matching. Our study investigated the impact of prior statin use on the clinical outcomes of critically ill patients with COVID-19. Additionally, we evaluated the statins' possible effects on the ICU-acquired complications, including safety outcomes. Most of the patients in the study cohort were given statins as part of their pre-admission medication regimen, with more than 80% of them receiving a moderate-intensity statin. The proportion of patients receiving statin was higher in our study than in previous reports (28, 29).

This study demonstrated that statin therapy in ICU patients with COVID-19 was associated with reduced risks of 30-day and all-cause in-hospital mortality. Despite the fact that statin users had a higher risk of developing severe COVID-19 due to a higher prevalence of co-morbid conditions, including diabetes mellitus and dyslipidemia, statins were found to have positive effects. This further supports the potential benefits of statins in COVID-19 management perhaps more specifically in subjects with pre-existing cardiovascular risk factors. When we divided the analysis by new commencement of statin treatment versus chronic statin use, the outcomes were in accordance with the primary analysis; nonetheless, they were not statistically significant. This benefit of statins might be attributed to its pleiotropic anti-inflammatory properties reducing the CRP levels and interleukin 6 (30). These inflammatory markers are known to increase during COVID-19 disease and increase the risk of mortality (30). In addition, statins have an antioxidant effect and improve endothelial dysfunction, which might help decrease the Cardiovascular events due to the hyper-coagulopathy status during the course of COVID-19 (30). Theoretically, statins may help reduce the cytokine storm which might be associated with the poor prognosis of patients infected with COVID-19 (18). Similar to our findings, a meta-analysis conducted by Kow et al. reported a significant reduction in severe COVID-19 disease with the use of statins (25). However, not all the included studies in this meta-analysis had critically ill patients in their study population (25). On the other hand, another meta-analysis by Scheen et al. and an observational study by Russo et al. showed that statins were not associated with mortality benefits in patients with COVID-19 (31, 32). This variation from our findings could be attributed to several reasons, such as differences in the population studied in the previous studies, and differences in the study methods used (27, 32). Contrary to our findings, a randomized controlled trial (INSPIRATION-S) presented in the American College of Cardiology (ACC 2021) comparing atorvastatin 20 mg daily versus placebo showed that initiating statin in critically ill patients with COVID-19 was not associated with mortality benefits (33). However, they included only the new initiation of statin therapy compared to our study in which most of our population were long-term statin users. This is comparable to the findings of our subgroup analysis, which revealed no differences in the study outcomes between the statin and control groups in participants who newly initiated on statin therapy. The prolonged anti-inflammatory effect of statins might significantly reduce the levels of inflammatory markers such as CRP; as a result, the suggested clinical benefit of statin therapy could emerge as early as 30 days after starting treatment, and it is consistent over time (19). Thus, prolonged statin use prior to admission might lessen the severity of cytokines storm and its complications. A study was conducted on patients with COVID-19 to assess the disease severity and mortality benefits between statin users (prolonged use) and non-statin users (28). Although, there were non-significant difference between the groups in terms of mortality benefits, the invistigators reported higher severity of COVID-19 in statin users which is contradicting our findings. It is important to note that our study was conducted on critically ill patients who are suffering from ARDS and severe inflammatory response in which continuing statin at peak of the inflammatory process might be of value in contrast with the mild or moderate disease who might not have ARDS or severe inflammatory response.

We found that the use of statins was not associated with any significant reduction in respiratory failures that required MV or longer VFDs. Contrary to our findings, a preliminary study in non-ICU COVID-19 patients demonstrated a significant reduction in the risk of MV in patients using statin (34). Another retrospective study that assessed the association between antecedent use of statin and COVID-19 outcomes in non-ICU patients found no significant association between statin use and the need for mechanical ventilation (*p* = 0.6) even though the investigators reported >30% ICU admission in their cohort (35). This may suggest the fact that the mortality benefit associated with statin therapy in our cohort was not primarily due to anti-inflammatory effects. Nonetheless, vasculoprotective and immunomodulatory effects could explain these benefits without reducing respiratory failure or MV needs.

Interestingly, patients who received statins had lower odds of pneumonia (bacterial or fungal). This finding is consistent with previous data, which could be related to a proposed antibacterial effect for statins (36–38). On the contrary, another study found that the prevalence of pneumonia in COVID-19 patients was similar between statin and non-statin users, with worse radiological features were confirmed after PS matching in the statin group (28). However, this study was not conducted on severe COVID-19 patients, and the worsening in radiographic features might be explained by increasing the severity of COVID-19 disease itself and not pneumonia as observed in their findings by increasing the National Early Warning Score (NEWS) (28). On the other hand, we observed that critically ill patients who received statins had significantly longer ICU and hospital LOS. The higher survival rate and the higher odds of secondary fungal infection in patients who received statin therapy could explain the prolonged ICU and hospital LOS in our cohort in the statins group. The higher rates of secondary fungal infections in the statin group are an interesting finding that has not been reported previously in COVID-19 patients. The prolonged ICU and hospital stays, rather than statin use, could be the major contributor of these infections. Early ICU admission, respiratory failure, and significant lymphopenia have all been documented to be risk factors for secondary infections in COVID-19 patients (39). In addition, various organisms have been related to secondary mucosal infections in COVID-19 patients, including C pneumoniae, human metapneumovirus, human parainfluenza virus, rhinovirus, enterovirus, and influenza B virus (40). However, due to retrospective observational design, examining subsequent mucosal infections and their causal factors were limited.

Besides the observed survival benefits in our study with statin use in critically ill patients with COVID-19, we did not observe any safety concerns related to statins' side effects or complications. Even though earlier reports from a cohort of 1,099 patients with COVID-19 from China showed that up to 39.4% had aspartate aminotransferase (AST) >40 U/L and 28.1% had Alanine aminotransferase (ALT) >40 U/L, most of these elevations occurred in critical COVID-19 cases (41). We did not find any significant difference in liver injury among the statins and non-statins critically ill groups with COVID-19. Statins showed a good safety profile in our cohort. Still, its use might be hindered by clinicians' reluctance to utilize it in critically ill patients with COVID-19 due to the fear of liver injury, myotoxicity, and rhabdomyolysis-related kidney injury. It is important to note that several reports portray the relationship between COVID-19 and rhabdomyolysis in critically ill patients (42–45). Thus, the cases of rhabdomyolysis in this cohort might have been related to COVID-19 rather than a result of statin use. Therefore, continuing statin therapy as primary or secondary prevention is advisable prior to ICU admission and during ICU stay unless contraindicated.

We believe that our multicenter cohort study is one of few studies that evaluated statins' clinical and safety outcomes in critically ill patients with COVID-19 using PS matching and multiple regression analysis to minimize the bias. Nevertheless, we also determined some limitations in our study. The retrospective nature of our study may have been affected by missing documentation that could be translated to unmeasured confounders. Also, the medication history before admission might be affected by limited reconciliation during COVID-19 pandemics. Thus, collecting data regarding the length of statin use prior to admission or data related to baseline lipid profile was limited. Furthermore, although we did not include a full list of the medications used in the ICU, we did report, and adjust for, the use of COVID-19 related medication including tocilizumab and corticosteroids. In addition, we included information on the use of nephrotoxic drugs, inotropes, vasopressors, and DVT prophylaxis during the ICU stay. Even though we observed mortality benefits, due to limited follow up period we were unable to assess statin intensity and the long-term benefit of statin use after COVID-19 survival. Furthermore, we were unable to evaluate each type of infection independently since we included both viral and bacterial infections in the same outcome category for pneumonia. Thus, a large randomized controlled trial is needed to investigate the efficacy and safety of statin use in critically ill patients with COVID-19.

## Conclusion

The use of statins during ICU stay in critically ill patients with COVID-19 was associated with lower mortality with no safety concerns. Thus, the use of statins in patients with COVID-19 during ICU stay might be a reasonable approach unless contraindicated. The result of this multicenter retrospective study motivates further prospective clinical studies to confirm our findings.

## Data availability statement

The raw data supporting the conclusions of this article will be made available by the authors, without undue reservation.

## Ethics statement

The study was approved in January 2021 by King Abdullah International Medical Research Center Institutional Review Board, Riyadh, Saudi Arabia (Ref. #NRC21R/015/R). Participants' confidentiality was strictly observed throughout the study by using anonymous unique serial numbers for each subject and restricting data only to the investigators. Informed consent was not required due to the research's method as per the policy of the governmental and local research center.

## Author contributions

All authors made a significant contribution to the work reported, whether that is in the conception, study design, execution, acquisition of data, analysis and interpretation, or in all these areas, took part in drafting, revising, or critically reviewing the article, gave final approval of the version to be published, have agreed on the journal to which the article has been submitted, and agree to be accountable for all aspects of the work.

## Funding

This study was supported by Princess Nourah Bint Abdulrahman University Researchers Supporting Project number (PNURSP2022R78), Princess Nourah Bint Abdulrahman University, Riyadh. Saudi Arabia.

## Conflict of interest

The authors declare that the research was conducted in the absence of any commercial or financial relationships that could be construed as a potential conflict of interest.

## Publisher's note

All claims expressed in this article are solely those of the authors and do not necessarily represent those of their affiliated organizations, or those of the publisher, the editors and the reviewers. Any product that may be evaluated in this article, or claim that may be made by its manufacturer, is not guaranteed or endorsed by the publisher.
