# Supplementary material for: The impact of HMG-CoA reductase inhibitors use on the clinical outcomes in critically ill patients with COVID-19: A multicenter, cohort study
Source: Front Public Health. 2022 Aug 11;10:877944. doi: 10.3389/fpubh.2022.877944 (PMC9403132; doi:10.3389/fpubh.2022.877944)
Supplement: Supplementary file 1 [file Data_Sheet_1.docx]

**Outcome definition (s)**

- The 30-day mortality was defined as a death occurring for any cause within 30 days of the admission date during a hospital stay; patients who were discharged from the hospital alive were presumed to be survived. For patients who died within the hospital stay but after 30-days of hospital admission, this outcome would be labeled as in-hospital mortality rather than 30-day mortality. All patients were followed until they were discharged from the hospital or died during the in-hospital stay, whichever occurred first.
- Ventilator-free days (VFDs) at 30 days were calculated as the following: if the patients die within 30 days of MV, the VFDs = 0, VFDs = 30 − days after MV initiation (if patient survived and was successfully liberated from MV), and VFDs = 0 if the patient is on MV for >30 days.
- Acute kidney injury (AKI) was defined as a sudden decrease of renal function within 48 hours, defined by an increase in absolute SCr of at least 26.5 μmol/L (0.3 mg/dL) or by a percentage increase in SCr ≥ 50% (1.5× baseline value) during ICU stay[24]_._
- Acute liver injury was defined as alanine aminotransferase (ALT) exceeding three times the upper limit of normal or double in patients with elevated baseline ALT during the ICU stay.
- Hospital-acquired pneumonia was defined as pneumonia that occurs > 48 hours after admission and did not appear to be incubating at the time of admission[25].
- Secondary fungal infection was identified through the blood, urine, wound, drainage, cerebrospinal fluid, and/or respiratory cultures. The fungal growth was considered significant if the growth was ≥ 100,000 colony forming units (CFUs)/ml in sputum or endotracheal aspiration, ≥10,000 CFUs of single organism/ml in bronchoalveolar lavage or ≥ 1000 CFUs of single organism/ml in protected specimen brushes. Additionally, urinary cultures were considered significant if showing a growth ≥100,000 CFUs/ml of no more than two species of microorganisms. Cultures were excluded if the laboratory reported them as a "contaminant sample."[26, 27]
- Respiratory failure was defined as either low arterial carbon dioxide tension (PaCO_2_) or hypoxemic respiratory failure (PaO_2_ < 60 mm Hg with a normal or hypercapnic respiratory failure (PaCO_2_ > 50 mm Hg) that requires mechanical ventilation.
